# Meeting Report VLPNPV: Session 10: Rapid total particle quantification

**DOI:** 10.4161/21645515.2014.986994

**Published:** 2014-12-15

**Authors:** Michael Artinger

**Affiliations:** ViroCyt, LLC; Boulder, CO USA

Mark Rehse form ViroCyt opened his talk with the question of how many viruses are in the ocean as a way of introducing the topic of virus quantification. As it turns out, there is an estimated 100 million viruses in a milliliter of your average sea water, which—if you do the math—means that there are 1.3 × 10^32^ viruses in all of the Earth's oceans. Add to this the fact that we have no idea how many different species of viruses there actually are. He then segued into the topic of virus quantification and that many of the current methods for quantifying viruses are outdated, expensive and highly subjective. For example, the plaque titer assay is essentially the same as it was when first used in 1952. Infectivity assays in general tend to be fairly slow, taking anywhere from 3–14 d to obtain results depending on the virus of interest. More recent technologies such as transmission electron microscopy are very costly, difficult to use and do not differentiate between empty and intact viral particles. So, how does this work in today's environment especially when someone is using emerging approaches such as Virus-Like Particles and what can we do to improve quantification? This is a problem that extends throughout many industries, especially during the production of viruses.

**Figure 1.**
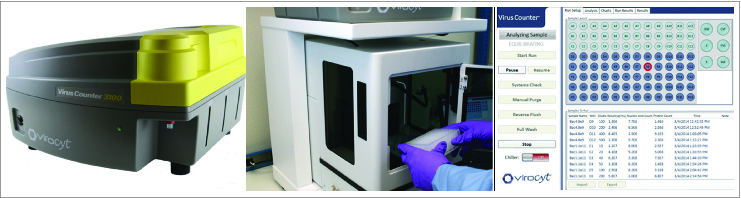
Virus Counter 3100 (left), autosampler (middle) and software (right).

Vaccine manufacturing was one the first applications that ViroCyt focused on improving processes by using true quantitative technologies. A significant amount of work has also been done with baculovirus expression systems, antiviral drug development, and more recently, viral therapeutics or so-called oncolytic virocides. It's a huge, growing industry in the United States, generally in teaching hospitals, where they have viral vector core labs and they’re taking retroviruses, vaccinia viruses—creating a variety of oncolytic viruses—by engineering them to deliver a message to a target in the solid tumor. Incredibly interesting stuff and very effective, but they all lack one thing, which is a new technology, a modern technology to accurately and quickly quantify viruses. As a result, ViroCyt created the Virus Counter®. The Virus Counter 3100 is the newest addition to ViroCyt's portfolio of products, and includes a standalone component as well as an optional 96-well plate autosampler (**Fig. 1**). Integrating the 2 produces an automated, walkaway virus quantitation platform. A full plate can be analyzed overnight, and the data evaluated the next morning. The system has been designed primarily for manufacturing settings, and thus, 21 CFR Part 11-capable software has been introduced to help ensure a GMP-controlled environment.

He then discussed how the Virus Counter technology works. It's basically a modified flow cytometer and as many of the attendees in the audience recognize, flow cytometers were originally designed to measure white blood cells, and generally particles 2 microns and larger. Where the Virus Counter comes in is below that range, all the way down to about 25 nanometers in size, which encompasses more than 90% of viruses, including Virus-Like Particles or VLPs. The other part of the system is the kit chemistry that was developed. The current kit is called Combo Dye and consists of 2 dyes: One dye labels nucleic acids, including both single- and double-stranded DNA and RNA. For some VLP products—those lacking nucleic acid payload—this can be an issue. The second dye, however, labels proteins. Many in the field use OD495 measurements for total protein content of the final product. The Virus Counter can provide essentially the same information and if there is genetic material, will also provide confidence that the result isn't just free protein, but an actual VLP particle. The dye kit works in combination with the flow cytometer to identify an array of about 10,000 events per second and the great thing about flow cytometry has always been statistical accuracy. Since the instrument is measuring tens of thousands of particles or more during a one minute analysis, it enables accuracy as well as reproducibility, something that traditional technologies—plaque assays, TCID50 and even PCR—suffer from. In addition, PCR can be problematic: Controls are required, as are primers and probes specific to the virus particle. The Combo Dye kit is also very affordable with a cost per test of less than $5, or roughly half of that of ELISAs for instance. By way of versatility, a wide assortment of viruses has been quantified on the Virus Counter (**Table 1**).

**Table 1.** Viruses quantified using Virus Counter

Arenavirus	HCV	Pox
Baculovirus	HSV	Rotavirus
BVD	IBR	RSV
CMV	Influenza	Rubella
Coronavirus	Lentivirus	Sendai
Coxackievirus	Measles	Vaccinia
Dengue	MVA	VEEV
Ebola	Parainfluenza	VLPs
Enterovirus	Paramyxoviridae	VSV
Hazara	Pichinde	Yellow Fever

**Figure 2.**
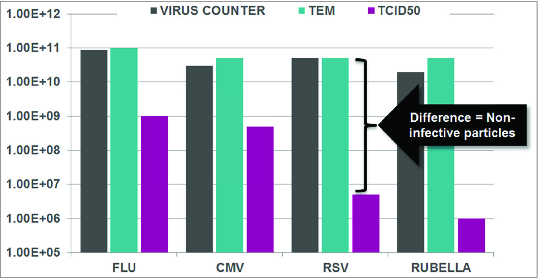
Relationship between Virus Counter, TEM and TCID50.

Another fundamental difference between infectivity assays and the Virus Counter is that the total particle information provided by the latter is the combination of infective and non-infective or so-called defective interfering particles. But why is this important? Depending on the virus of interest, the particle to pfu ratios are anywhere from 1:1 to as much as 1:10,000, meaning that for every 10 thousand particles, only one is infective (**Table 2**). The challenge for anyone in a manufacturing environment is when they produce a vial of final product, do they really know with 100% certainty what is in it? If they are only looking at infectivity, there is a huge defective particle component, which as many people know, are immunogenic. So, it's important to understand exactly what is in the sample. The Virus Counter provides the total viral particle concentration, and if this is combined with pfu studies, you have a very powerful description of the final product. In other words, the total particle to pfu ratio is essential to every measurement product profile in the industry. **Figure 2** further illustrates this point. Comparing Virus Counter results with transmitting electron microscopy data demonstrates a very close correlation, which would be expected since both quantify viral particles. Where the coincidence of absolute values isn't as close is with infectivity results. The absolute number, however, is a floating number: What does it actually mean? The essential value is correlation: How well do the Virus Counter or TEM results associate with growth patterns of viruses? In actuality, these correlate quite well and this is an important point since it means that viral particle counts can be used to make critical decisions about when to harvest, what conditions to grow virus particles in, which construct has the highest productivity, and so on. Those are decisions that need to be made quickly because we can't afford to wait 3 to 14 d for plaque titer assays to make these kinds of decisions in the manufacturing environment. The Virus Counter offers a powerful alternative to outdated methods.

**Table 2.** Particle to infectivity ratio for different viruses

Adenovirus	20–100
Alphavirus	1–2
Herpes Virus	50–200
Influenza	20–50
Papillomavirus	10,000
Polio	30–1,000
Polyomavirus	38–50
Simian Virus 40	100–200
Pox	1–100
Reovirus	10

Mark then discussed current users of the Virus Counter, which include both domestic and international companies, institutes, agencies and universities, such as Sanofi Pasteur, Protein Sciences, GE Healthcare, the National Institutes of Health, the United State Food and Drug Administration and the University of Maryland. The instrument has also been adopted by the Filovirus Animal Nonclinical Group (FANG), an interdepartmental and interagency group established to support and facilitate the creation of vaccines and therapies for Ebola and Marburg viruses. It is co-led by the Department of Defense and the Department of Health and Human Services, and includes participation by the governments of Canada and Great Britain.

Taking closer look at the instrument, the software interface for the autosampler coupled system indicates which well is currently being analyzed, which wells have been completed as well as those that have been programmed for quantification during the sequence. In the automated system, the liquid handling for both supply and waste has also been scaled up permitting up to 15 hours of continuous operation.

He went on to present several case studies as specific examples of how the Virus Counter is being used today, the first focusing on baculovirus. The applications of baculovirus—since it was first developed as an expression system in the early 1980s—have grown tremendously and it is now used for all sorts of expression vector programs and in final product manufacturing. The Virus Counter fits quite well into many aspects of the baculovirus system, such as the expansion phase, purification, and even in the determination of which construct produces the highest titer. These are at least 3 steps that can benefit from a way to measure total virus construct. Recently acquired data from a clinical site in the United States looked at 2 different types of baculovirus constructs: One designed to express GFP and the other AAV. There were 2 different concentrations of each of the constructs which were followed over a 60 hour growth period, and there is a dramatic difference between the AAV-producing component and the GMP-expressing component in terms of growth output. Mark then asked, “How many samples would it take to generate this data using traditional plaque titer assays? With the answer being quite a while. It raises the question of do we really want to spend the time, energy and money to do this, or should we just choose one option and go with it?” Using the Virus Counter provides the advantage of being able to make a decision on a number of constructs simultaneously, since there could have been 3 or 4 or 5 more constructs evaluated and take the same amount of time to generate the data.

Mark then mentioned that the staining protocol takes 30 minutes at room temperature in the dark. The dyes are intercalating equilibrium dyes and not antibody-based, so there is no wash step involved, and one minute to acquire the data.

**Figure 3.**
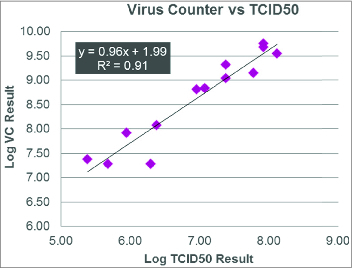
Comparing Virus Counter and TCID50 results for multiple baculovirus samples.

In the next use example, a set of 13 different baculovirus constructs were evaluated using TCID50 and the Virus Counter (**Fig. 3**). Typically, a correlation of 0.95 between the 2 methods and a slope in this case of positive 1.0 is a good indication of the accuracy between the expected and measured values. This is a useful tool to determine a number of growth parameters, including MOIs for the effective infectivity rate, and allows quick decisions to be made about which construct to choose based on which has the highest growth rate to move forward in the production process. Also discussed was the evaluation of P0 and P1 ratios, which construct had the best MOI and therefore the highest productivity given a unique set of environmental conditions. This is another experiment easily done with the Virus Counter. If one were to put error bars on the bar chart, we would generally see a Coefficient of Variation (CV) of 15% or less. So, there is very good reproducibility with the system as well, especially since it's automated.

**Figure 4.**
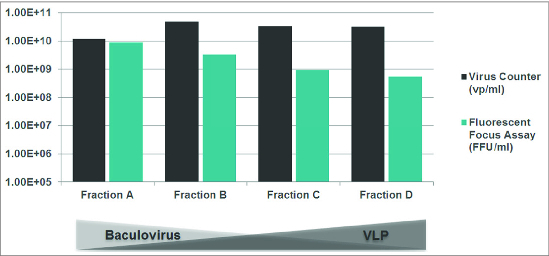
Removal of baculovirus relative to VLPs during purification.

**Figure 5.**
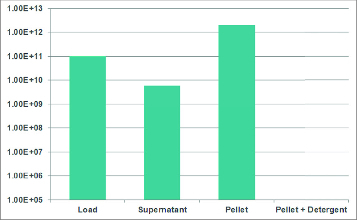
Tracking VLPs at each step of an ultracentrifugation procedure.

VLPs were the focus of the next group of results. Since VLPs are non-infective, many traditional approaches to quantification are not useful. As mentioned previously, the Virus Counter can replace OD495 measurements and is much faster and more reproducible. In work carried out by a collaborator, the enrichment of VLPs generated by a baculovirus expression system was tracked using Virus Counter and the Fluorescent Focus Assay (**Fig. 4**). Because the baculovirus component is infectious, one can see that-during the purification process-the Fluorescent Focus Units decrease while the total particle count increases or plateaus. This strongly suggests that the VLPs are fractioning into the centrifugation product while the baculovirus is spun down and separated out. A second series of experiments diagrammed an ultracentrifugation experiment comparing the supernatant and pellet for VLP content (**Fig. 5**). Taking the initial mixture, pre-centrifugation, and looking at total particle content using the Virus Counter, provides a result of approximately 1×10E11 vp/ml. Separating out the supernatant from the pellet post-centrifugation has the particle count decrease to 8 × 10E9 vp/ml in the supernatant while the pellet is enriched to 2 × 10E12 vp/ml. The addition of detergent eliminates all counts, providing additional proof that the particles are indeed VLPs.

In summary, the Virus Counter requires a minimum sample volume of 100 microliters, has a detection limit of 1×10E5 to 1×10E9 vp/ml, and the upper limit can be addressed using serial dilution of concentrated samples. The total analysis time is 6 minutes, but this can be decreased depending on the nature of the samples. It takes 1 minute to quantify the actual sample, followed by an intersample wash using a low pH solution and verification that there isn't any residual carryover from the previous sample.

